# Boosting the Interfacial Stability of the Li_6_PS_5_Cl Electrolyte with a Li Anode via In Situ Formation of a LiF-Rich SEI Layer and a Ductile Sulfide Composite Solid Electrolyte

**DOI:** 10.1021/acsami.3c14763

**Published:** 2024-02-15

**Authors:** Gashahun Gobena Serbessa, Bereket Woldegbreal Taklu, Yosef Nikodimos, Nigusu Tiruneh Temesgen, Zabish Bilew Muche, Semaw Kebede Merso, Tsung-I Yeh, Ya-Jun Liu, Wei-Sheng Liao, Chia-Hsin Wang, She-Huang Wu, Wei-Nien Su, Chun-Chen Yang, Bing Joe Hwang

**Affiliations:** † Nano-electrochemistry Laboratory, Department of Chemical Engineering, National Taiwan University of Science and Technology, Taipei City 106, Taiwan; ‡ Nano-electrochemistry Laboratory, Graduate Institute of Applied Science and Technology, National Taiwan University of Science and Technology, Taipei City 106, Taiwan; § Battery Research Center of Green Energy, Ming-Chi University of Technology, New Taipei City 24301, Taiwan; ∥ Department of Chemical Engineering, Ming Chi University of Technology, New Taipei City 24301, Taiwan; ⊥ National Synchrotron Radiation Research Center (NSRRC), Hsinchu 30076, Taiwan; # Sustainable Electrochemical Energy Development (SEED) Center, National Taiwan University of Science and Technology, Taipei City 106, Taiwan

**Keywords:** solid-state battery, lithium metal anode, in situ LiF generation, dendrite suppression, interfacial stability, solvent-free solid sulfide composite electrolyte

## Abstract

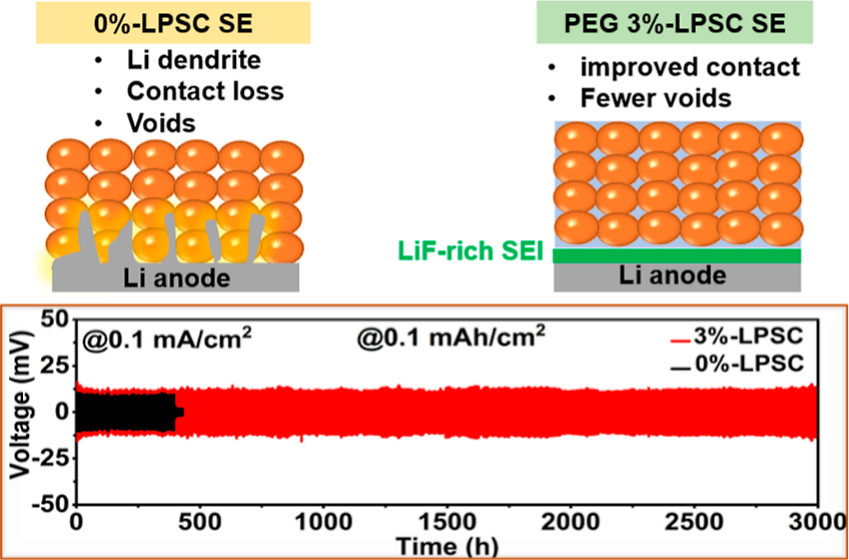

Due to its good mechanical properties and high ionic conductivity, the sulfide-type solid electrolyte (SE) can potentially realize all-solid-state batteries (ASSBs). Nevertheless, challenges, including limited electrochemical stability, insufficient solid–solid contact with the electrode, and reactivity with lithium, must be addressed. These challenges contribute to dendrite growth and electrolyte reduction. Herein, a straightforward and solvent-free method was devised to generate a robust artificial interphase between lithium metal and a SE. It is achieved through the incorporation of a composite electrolyte composed of Li_6_PS_5_Cl (LPSC), polyethylene glycol (PEG), and lithium bis(fluorosulfonyl)imide (LiFSI), resulting in the in situ creation of a LiF-rich interfacial layer. This interphase effectively mitigates electrolyte reduction and promotes lithium-ion diffusion. Interestingly, including PEG as an additive increases mechanical strength by enhancing adhesion between sulfide particles and improves the physical contact between the LPSC SE and the lithium anode by enhancing the ductility of the LPSC SE. Moreover, it acts as a protective barrier, preventing direct contact between the SE and the Li anode, thereby inhibiting electrolyte decomposition and reducing the electronic conductivity of the composite SE, thus mitigating the dendrite growth. The Li|Li symmetric cells demonstrated remarkable cycling stability, maintaining consistent performance for over 3000 h at a current density of 0.1 mA cm^–2^, and the critical current density of the composite solid electrolyte (CSE) reaches 4.75 mA cm^–2^. Moreover, the all-solid-state lithium metal battery (ASSLMB) cell with the CSEs exhibits remarkable cycling stability and rate performance. This study highlights the synergistic combination of the in-situ-generated artificial SE interphase layer and CSEs, enabling high-performance ASSLMBs.

## 1 Introduction

In the pursuit of high-energy-density batteries, the utilization of a lithium anode is indispensable due to its remarkable characteristics, which include a high theoretical specific capacity (3.860 A h g^–1^), low density (0.534 g cm^–3^), and the most negative electrochemical potential (−3.04 V vs standard hydrogen electrode).^1–4^ Diverse liquid electrolyte (LE) systems have been utilized to achieve extended cycling of lithium metal anodes. However, the uneven deposition of lithium metal in these systems can result in the formation of hazardous lithium dendrites.^5^ These dendrites pose significant safety risks in lithium metal batteries (LMBs) that utilize LEs, including the potential for short circuits, inflammation, and even explosions within the battery.^2,6,7^ To address this challenge, there is a growing expectation that solid-state electrolytes (SSEs) having high mechanical modulus and exceptional thermal stability will surpass LEs in LMBs, offering superior safety and high energy density.^8,9^ Due to its prominent characteristics, Li_6_PS_5_Cl (LPSC, Li-argyrodite) has attracted considerable attention among these solid electrolytes (SEs). These include a high ionic conductivity of over 1 mS cm^–1^ at room temperature, ease of synthesis, and the ability to be processed at low temperatures. These properties make it highly desirable for the large-scale production of solid-state batteries, offering the potential for scalable manufacturing processes.^10,11^ All-solid-state batteries (ASSBs) with a Li metal anode emerge as the most viable substitute to provide high energy density.^11–14^

Despite their potential advantages, it has been observed that most sulfide-based SEs cannot effectively suppress the growth of lithium dendrites.^1,15–18^ Theoretical calculations indicate that all sulfide-based SEs generally exhibit a relatively limited electrochemical stability window, typically ranging from 1.7 to 2.4 V.^19–21^ This constrained stability range gives rise to electrochemical reduction during the cell’s cycling, which, in turn, leads to the creation of undesirable interfacial reduction byproducts.^1,22^ The high electronic conductivity and presence of grain boundaries (GBs), holes, and cracks in SEs are likely contributing factors to the generation and growth of lithium dendrites.^1,16,23,24^ Lithium dendrites develop and grow within regions rich in defects, such as GBs and pores in SSEs.^25,26^ Thus, owing to the poor adhesion among sulfide particles, the resistance for dendrite growth is diminished in sulfide SEs, leading to the evident formation of dendrites.^27^ Conversely, the poor interface contact between the SE and lithium metal can result in the inhomogeneous deposition of lithium. It can exacerbate the propagation of lithium dendrites and fail ASSLBs.^8,12,23,28^

Hence, developing innovative sulfide-based electrolytes that exhibit high ionic conductivity and enhanced interfacial properties is critical. Composite SEs (CSEs), designed by blending sulfide glass and a polymer component, show great potential for improving the safety of ASSLBs. These composite electrolytes provide the merits of sulfide-glass-based and polymer electrolytes, making them highly desirable for battery applications.^10,29^ Polymer–inorganic composite electrolytes offer several advantages in improving the contact between electrodes and buffering volume changes during the lithium plating/stripping.^12^ This improved contact promotes efficient ion transport across the interface and reduces interface resistance, enhancing the overall battery performance. This feature also plays a significant role in preventing direct contact between unstable sulfide SEs and lithium metal, thus bolstering the overall stability of the electrolyte.^30,31^

The synthesis of sulfide-based CSEs entails mixing binders and sulfide SEs through a solvent-based slurry process to achieve the desired composite electrolytes.^32^ However, due to the reactivity of sulfide-based SEs with common solvents used to dissolve polymers, their ionic conductivity decreases significantly when dispersed and dried in those solvents.^22,29^ Hence, choosing an appropriate solvent poses a significant challenge when developing new sulfide-based composite SEs.^10^ Therefore, adopting an innovative and unconventional approach is crucial to overcoming the limitations of the slurry-based strategy for processing sulfide-based SSEs.

An alternative, highly efficient approach to mitigate undesired side reactions and suppress the formation of lithium dendrites is establishing a durable solid–electrolyte interface (SEI) layer between the SEs and the lithium metal anodes.^33,34^ This can be achieved via a spontaneous chemical reaction on the surface of the Li anode during the initial cycling stages. To impede the growth of lithium dendrites, it is essential to design an electron-insulating layer that exhibits high interfacial energy for lithium.^1,35^ Chen et al. successfully suppressed the development of Li dendrites by creating an SEI enriched with LiF through a sustained release effect.^26^ Simon et al. employed a stable solid polymer electrolyte as a protective interlayer to prevent the direct contact and reaction of LPSC SE with the lithium metal anode.^36^ Liu et al. established a compact interface enriched with Li_3_N in situ between the N-doped LPSC electrolyte and the lithium metal anode.^19^ Zou et al. created a robust SEI layer at the Li/LPSC interface through the electrochemical reduction of the LiTFSI/tetraethylene glycol dimethyl ether LE by assembling a pseudo-solid-state battery using ionic liquids.^37^ Fan et al. pioneered the creation of an SEI enriched with LiF through the interaction of lithium with lithium bis(fluorosulfonyl)imide (LiFSI)-coated or infiltrated LPS.^38^ Nevertheless, the aforementioned interfacial modification techniques often have high costs and complexity. None of these approaches have harnessed the synergistic effect of a composite solid sulfide electrolyte combined with the in situ generation of a LiF-rich SEI to mitigate dendrite growth effectively.

Herein, our study focuses on designing and successfully showcasing a novel approach to overcome these challenges and create a durable SEI. We have developed a solvent-free sulfide-incorporated composite electrolyte synthesis method and the in situ construction of a LiF-rich SEI. The solvent-free sulfide composite electrolyte preparation was possible using a liquid poly(ethylene glycol) (PEG) additive at room temperature. In addition, the in situ creation of a LiF-rich SEI was enabled through the introduction of LiFSI. This choice was based on LiFSI’s strong affinity for reacting with Li metal, leading to the development of a stable LiF-rich layer on the surface of the Li metal during the lithium plating/stripping process.^38^ A LiF-rich SEI boosts even the deposition of Li, suppressing dendrite growth and preventing penetration into SSEs. The LiF-rich SEI layer also inhibits side reactions, enhancing stability. The composite electrolyte and LiF-rich SEI synergistically improve the critical current density (CCD) (exceeding 4.75 mA cm^–2^) and enable above 3000 cycles at 0.1 mA cm^–2^ for Li|Li symmetric cells.

## 2 Experimental Section

### 2.1 Material Preparation

#### 2.1.1 Preparation of PEG/LiFSI Slurry

PEG (average MW, 400) and LiFSI (99.9%, Alfa Aesar) were used to prepare the PEG/LiFSI slurry in a molar ratio of 10:1 (EO/Li).^39–41^ Before use, the salt was dried under vacuum at 80 °C for 24 h. Additionally, the slurry was stirred in a glovebox for 24 h at 25 °C.

#### 2.1.2 Preparation of the LPSC Composite SSE

To prepare *x*%-LPSC powder (1 g), the weight percentage (wt %) ratio of the commercial SSE LPSC ( NEI Corporation) and the as-prepared PEG/LiFSI slurry was measured. Then, the resulting mixture was transferred to a mortar and pestle and manually ground for 30 min. The synthesized solid sulfide composite electrolytes were named *x*%-LPSC (where *x* = 0, 1, 2, 3, 5, and 10) based on the PEG/LiFSI wt % composition. After preparation, the *x*%-LPSC CSEs were stored in a glovebox for future experimentation. X-ray diffraction (XRD) and Raman spectroscopy tests were conducted on both freshly prepared and 45 day old samples of the CSEs to confirm the long-term chemical stability between the LPSC SE and the PEG polymer.

#### 2.1.3 LCO Composite Cathode Preparation

To assemble ASSLMBs, the composite cathode was synthesized by blending LiCoO_2_ (LCO), Li_3_InCl_6_ (LIC), and vapor-grown carbon fiber (VGCF) in a weight ratio of 70:27:3, respectively. The mixture was then transferred to a mortar and pestle and hand-milled for 30 min using an agate mortar. The VGCF was subject to a 24 h drying process in a vacuum oven at 80 °C. The experiments were conducted within a glovebox filled with argon (Ar) gas, ensuring that the levels of oxygen (O_2_) and water (H_2_O) were kept below 0.1 ppm.

### 2.2 Material Characterization

A field-emission scanning electron microscope (JEOL JSM-6500 F) was utilized to analyze the surface morphology of the Li metal anode after cycling as well as the SEs before and after cycling. The elemental distribution of the as-prepared CSE was determined by using energy-dispersive X-ray spectroscopy (EDX). The Bruker D2 Phaser diffractometer, utilizing a Cu Kα radiation source (λ = 1.5406 Å), was employed to analyze the crystalline phase of the CSEs. The 2-theta range for the scan extended from 10 to 80°. Raman measurements were executed to investigate the vibrational modes of the thiophosphate group (PS_4_^3–^), by using a Uni-Ram Raman spectrometer. The excitation laser wavelength employed for the measurements was 532 nm. During the measurements, the samples were placed inside transparent glass containers and sealed with air-tight glue to protect them from exposure to air. In addition, X-ray photoelectron spectroscopy (XPS) measurements were performed at the beamline station BL 24A1, situated at the National Synchrotron Radiation Research Center (NSRRC), Hsinchu, Taiwan. The energy calibration of the entire XPS spectra was performed using the Au 4f_7/2_ peak as a reference at 84.1 eV.

### 2.3 Electrochemical Characterization and Air Stability Measurement

#### 2.3.1 Electrochemical Impedance Spectroscopy

The VMP3 potentiostat SAS impedance analyzer (Bio-Logic) was used to take the AC impedance measurements at 10 mV, using stainless steel (SUS) as a blocking electrode within a frequency range of 10 MHz to 1 Hz. DC polarization measurements at different voltages were used to measure the electronic conductivities of samples using SUS as the blocking electrode. Equation 1 was used to calculate the Li^+^ ion conductivities.
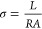
1where *L* is the electrolyte pellet thickness, *R* is the resistance of the electrolyte, and *A* denotes the area of the SSE pellet.

#### 2.3.2 Cell Fabrication and Electrochemical Measurements

A Li|*x*%-LPS|CLi symmetric cell configuration was fabricated and operated at an areal capacity of 0.1 mA h cm^–2^ and a current density of 0.1 mA cm^–2^ to examine the compatibility of the SEs with the Li metal anode. A CR-2023 type coin cell was used to assemble a pellet with a 10 mm diameter on which a Li foil with an 8 mm diameter was affixed. Furthermore, the Li|3%-LPSC|Li symmetric cell was subjected to testing at elevated current densities of 0.5, 1, and 2 mA cm^–2^, employing a KP cell for the tests. Meanwhile, a Li|*x*%-LPSC|Li symmetric cell was also constructed to assess the interfacial stability of the SEs with the Li metal anode at different storage times. The CCD was investigated using a fixed step size of current density increment, set at 0.25 mA cm^–2^ for each cycle. A galvanostatic computer-controlled 40-channel battery tester (Arbin BT-2000, USA) was used to operate the cell within a potential range of +1 to −1 V.

To study the impact of PEG/LiFSI on the voltage stability window of the LPSC SE, the Li|3%-LPSC|SUS semi-blocking cells were used. An electrolyte pellet was formed by compressing 70 mg of 3%-LPSC powder under a pressure of 350 MPa. Then, the Li foil was affixed to the opposite side of the pellet, and the cell was assembled by using a CR-2023 type coin cell. The test was conducted by sweeping the voltage at a rate of 0.1 mV s^–1^ within the range of −0.5–5 V.

#### 2.3.3 ASSLMB Assembly

The assembly used 70 mg of *x*%-LPSC SE and 20 mg of LIC as an interlayer. Additionally, 10 mg of the as-prepared composite cathode was dispersed uniformly on the side of the LIC electrolyte as an interlayer, and the Li metal anode with an 8 mm diameter was placed on the other side. The SUS foil was employed as the current collector to sandwich the final battery. The cell was assembled by using a KP cell, and an external pressure of 5 N m was applied to ensure optimal contact and stability. The galvanostatic charging/discharging of the cells was conducted at 30 °C using an Arbin BT-2000 system. The cutoff voltages were set to 2.5–4.2 V (vs Li/Li^+^) during testing.

#### 2.3.4 Moisture Stability Testing

For the measurement, pelletized samples weighing 80 mg were carefully positioned in a sealed container with a volume of 1000 cm^3^. The container had a microfan and an H_2_S gas sensor (GX-2009, Riken Keiki Co., Ltd., Tokyo). Then, it was filled with air at a relative humidity of 20%, and the measurements were conducted at room temperature. A mobile video recorder captured the readings on the gas sensor screen for 30 min. The amount of H_2_S detected during this period was recorded.

#### 2.3.5 Mechanical Strength Measurement

To perform the tensile test and create a stress versus strain diagram, 100 mg of SE powder was employed to produce a pellet-type SE with a diameter of 10 mm. This was achieved through cold pressing with a pressure of 350 MPa for 3 min. The test was conducted in a dry room (dew point: ∼−40 °C). The EZ Test apparatus (Shimadzu-EZ-SX) was utilized, utilizing its rough surface holder to securely grasp the upper and lower edges of the pellet at a depth ranging from 3 mm to 4 mm. The stretching velocity was set at 20 mm/min.

## 3 Results and Discussion

### 3.1 Structural Characterization

**Scheme 1:**
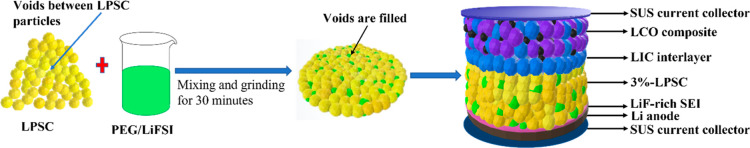
Schematic Illustrations of *x*%-LPSC Composite Sulfide SE Synthesis and ASSLMBs Configuration Using 3%-LPSC SE and Li Metal as the Electrolyte and Anode, Respectively

The systematic procedures used to prepare the composite sulfide SE and ASSLMB cell assembly are demonstrated in Scheme 1. As illustrated in the scheme, initially, there were gaps between the sulfide particles of the LPSC SE. However, the gaps were filled when these particles were mixed with the PEG/LiFSI slurry. Furthermore, during the initial cycling process, an SEI layer rich in LiF was created on the surface of the lithium metal anode.

**Figure 1:**
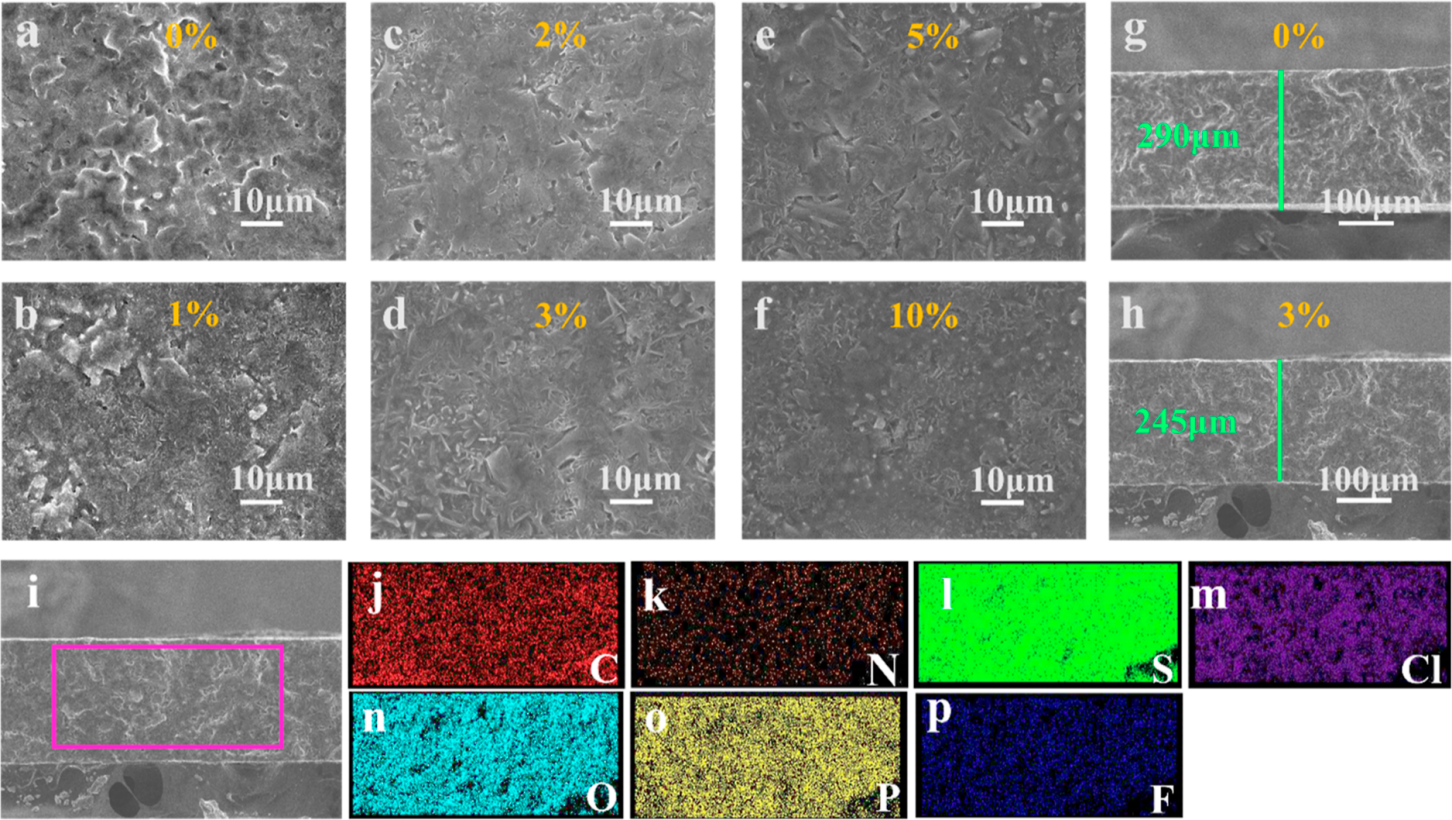
SEM images of (a) 0%-LPSC, (b) 1%-LPSC, (c) 2%-LPSC, (d) 3%-LPSC, (e) 5%-LPSC, and (f) 10%-LPSC CSEs. Cross-sectional SEM images of (g) 0%-LPSC and (h) 3%-LPSC SEs. (i) 3%-LPSC CSE cross-sectional view with its corresponding EDS elemental mapping (j–p) of C, N, S, Cl, O, P, and F elements, respectively.

SEM analysis was conducted to examine the morphology of *x*%-LPSC electrolyte pellets produced through cold pressing, as shown in Figure 1a–f. As depicted in Figure 1a, the surface characteristics of the LPSC SE powders exhibit inadequate adhesion between particles and a rough texture marked by numerous microvoids. These gaps could provide space for Li dendrites’ formation, leading to the possibility of short circuits.^25,26^ By incorporation of the PEG polymer, the LPSC particles underwent a gradual coating process. This also resulted in enhanced compactness of the sulfide particles, and the degree of compactness increased proportionally with the PEG content. Furthermore, the PEG polymer filled the gaps between the LPSC particles, facilitating the smooth transportation of Li^+^ ions and providing electronic insulation at the GBs. The results indicate that a PEG content of 3 wt % resulted in complete coverage of all LPSC particles, as shown in Figure 1d. It should be emphasized that in addition to filling the gaps between LPSC particles, PEG also filled the GBs, which played a critical role in impeding electron transport at GBs.^42^ The cross-sectional images in Figure 1g,h reveal that the 3%-LPSC SE pellet has a thickness that is thinner than that of the 0%-LPSC SE pellet. This difference suggests that the 3%-LPSC SE is more densely packed than the 0%-LPSC SE, attributed to the effective filling of voids by the PEG/LiFSI additives. Additionally, the bulk density of each pellet was calculated based on its weight and physical dimensions, which was found to be 1.53 g cm^–3^ for the 0%-LPSC and 1.838 g cm^–3^ for the 3%-LPSC SE. Subsequently, the relative density value was determined by dividing the bulk density by the theoretical density of LPSC (∼1.86 g cm^–3^),^43,44^ which is 82.25% for the 0%-LPSC and 98.81% for the 3%-LPSC SE pellets. These results indicate an improvement in density due to a decrease in voids in the 3%-LPSC SE. Both the EDS mapping images of the plane (Figure S1) and the cross section of 3%-LPSC (Figure 1j–p) demonstrated an even distribution of C, N, S, Cl, O, P, and F. This indicates that PEG/LiFSI was evenly dispersed within the LPSC structure. It should be emphasized that a higher PEG content would negatively impact the ionic conductivity, as illustrated in Figure 3a, due to the incorporation of the low ionic conductivity PEG polymer. This, in turn, would increase voltage polarization during cycling (as shown in Figures 4a and S8) and subsequently decrease the cycle life.

**Figure 2:**
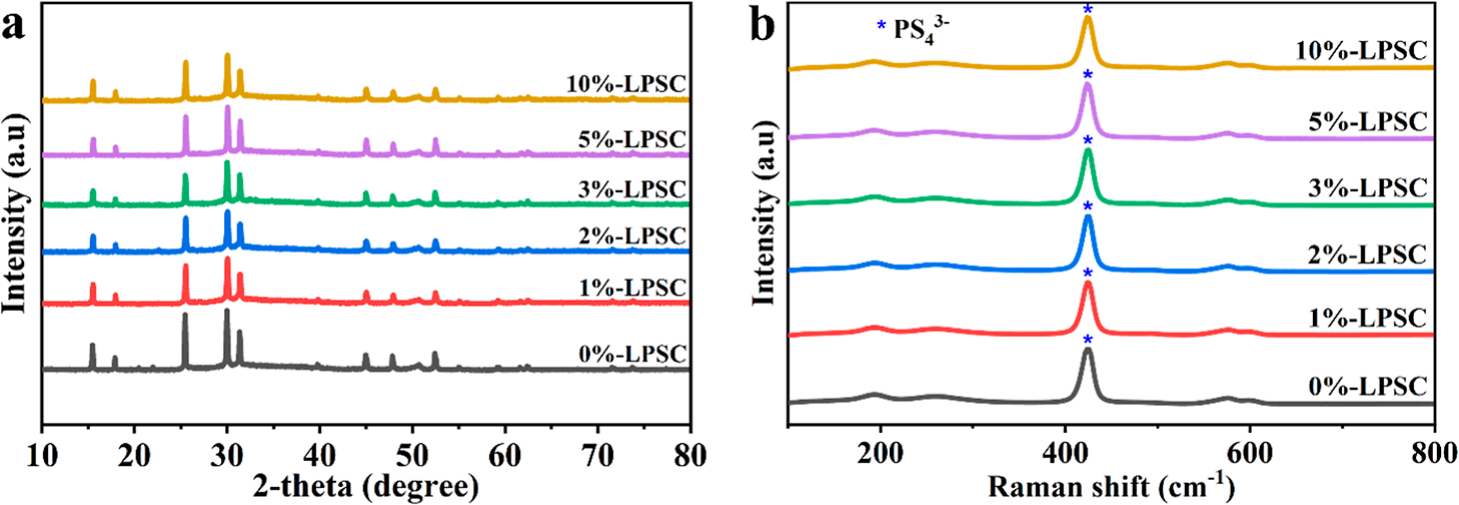
(a) XRD patterns and (b) Raman measurement for *x*%-LPSC electrolytes, where *x* = 0,1, 2, 3, 5, and 10 wt % of PEG/LiFSI.

**Figure 3:**
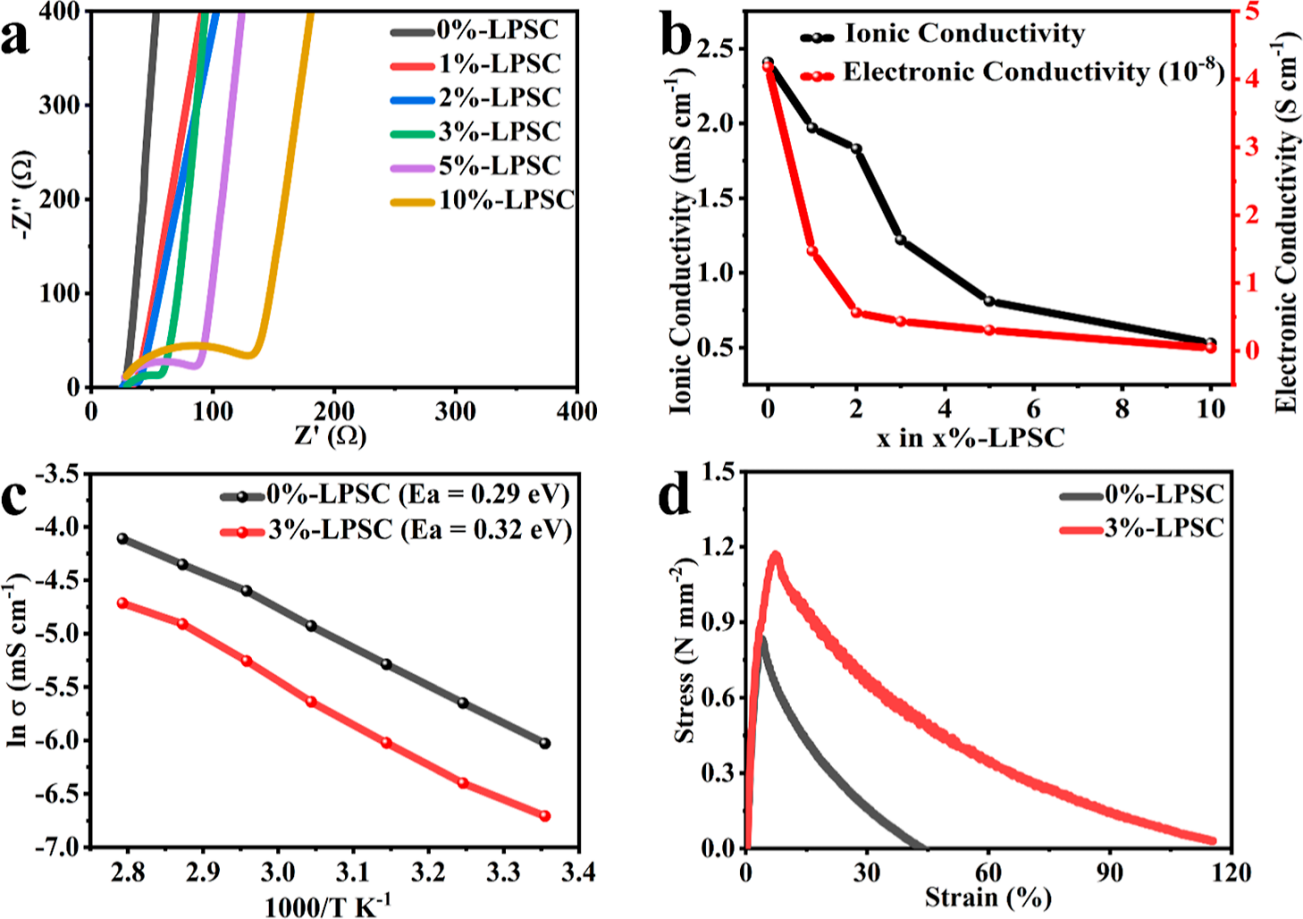
(a) Nyquist plot of 0%-LPSC, 1%-LPSC, 2%-LPSC, 3%-LPSC, 5%-LPSC, and 10%-LPSC SEs, (b) ionic and electronic conductivity comparison of *x*%-LPSC SEs at 25 °C, (c) Arrhenius plot of 0%-LPSC and 3%-LPSC SEs, and (d) stress–strain curves of 0%-LPSC and 3%-LPSC SEs.

**Figure 4:**
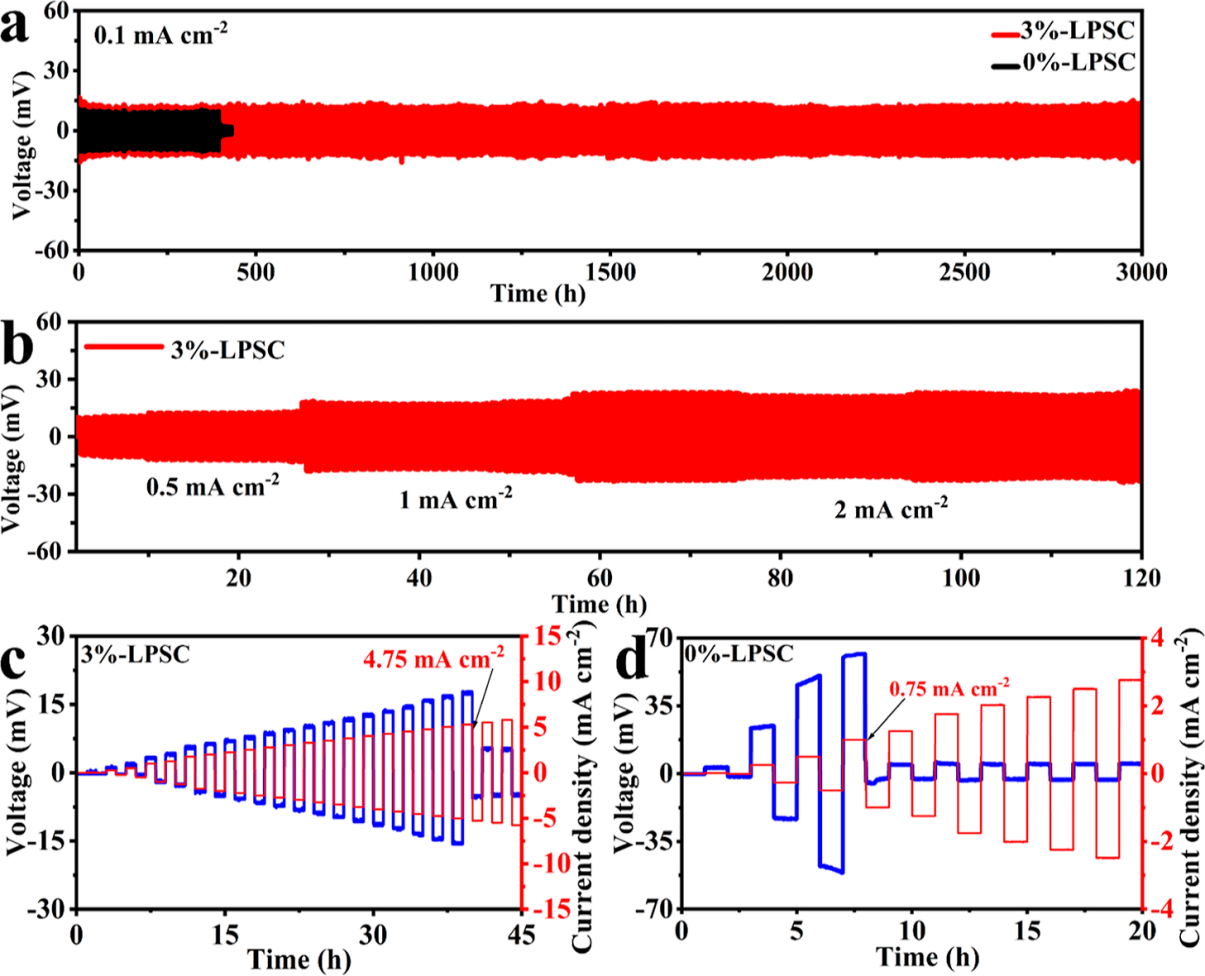
(a) Li|Li symmetric cell test for *x* = 0 and *x* = 3 SEs at a current density of 0.1 mA cm^–2^ and an areal capacity of 0.1 mA h cm^–2^ (coin cell). (b) Li|Li symmetric cell test for 3%-LPSC SE at different current densities (KP cell). Galvanostatic cycling of Li–Li symmetric cells (c) 3%-LPSC and (d) 0%-LPSC SEs at step-increased current densities.

The structural characteristics of the synthesized composite sulfide SE were analyzed by Raman spectroscopy, calibrated via a Si wafer, and by XRD to investigate the impact of incorporating PEG/LiFSI on the LPSC structure. Figure 2a illustrates the XRD patterns of *x*%-LPSC (where *x* = 1, 2, 3, 5, and 10), which were similar to that of 0%-LPSC. The Li-argyrodite phase displays clear and distinctive peaks at 2-theta angles of 26.35, 30.24, and 32.12°, consistent with findings reported in earlier studies.^32^ The absence of reflections other than those corresponding to the argyrodite structure suggests that LPSC retains its crystallinity while forming an *x*%-LPSC composite electrolyte. Additionally, Figure S2 illustrates the XRD patterns of the LiFSI salt and PEG polymer additives used for the composite solid sulfide electrolyte preparation. In Figure 2b, the Raman spectra of the CSEs that were prepared are displayed within the frequency range of 100–800 cm^–1^. Despite the addition of PEG/LiFSI, the position of the most prominent peak at 421 cm^–1^, which corresponds to the symmetric stretching vibration mode of the P–S bond in PS_4_^3^^,^^45^ remained constant. This result indicates that incorporating PEG/LiFSI into the argyrodite system has no impact on the local structure of the PS_4_^3–^ unit and does not alter the P–S bonds. Hence, it is presumed that the fundamental structure of the LPSC argyrodite system remains consistent in the CSEs. Consequently, PEG/LiFSI can potentially be a favorable candidate for developing LPSC-based solid sulfide composite electrolytes.

Additionally, to verify the long-term stability between the LPSC SEs and the PEG polymer, structural characterization was carried out using XRD and Raman tests after storing for 45 days (Figure S3). The results indicate that no peak shifts or disappearances were observed, which suggests high chemical stability between PEG and LPSC.

### 3.2 Electrochemical Performance

The ionic conductivities of different *x*%-LPSC composites were assessed (Figure 3a, Nyquist plot) and compared (Figure 3b). Due to the incorporation of low-ionic-conductive PEG, the ionic conductivity of *x*%-LPSC decreased with increasing PEG content. The ionic conductivity of *x*%-LPSC decreased noticeably from 2.4 to 0.53 mS cm^–1^ as the PEG/LiFSI content was increased from 0 to 10 wt %. The temperature-dependent electrochemical impedance spectroscopy (EIS) measurement was conducted across a temperature range from 25 to 85 °C. An Arrhenius plot, presented in Figure 3c, was utilized to determine the activation energy (*E*_a_) of these electrolytes using eq 2. For the 0%-LPSC electrolyte, an activation energy of 0.29 eV was obtained, which aligns with findings from a previous study,^32^ and for the 3%-LPSC pellet it is found to be *E*_a_ = 0.32 eV. The Nyquist plots of the impedance of *x*%-LPSC during ionic conductivity testing at various temperatures are displayed in Figure S4.
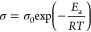
2where σ is the ionic conductivity, σ_0_ is the pre-exponential factor, *R* is the universal gas constant, and *T* is the temperature (in kelvin).

The nucleation and evolution of lithium dendrites within solid-state batteries are key factors contributing to cell failure. The presence of dendrites in SEs and their decomposition products at the electrode interface has been associated with increased electronic conductivity (σe) of the SEs.^23,46^ The electronic conductivity of SEs, along with their efficient Li^+^ ion transport characteristics, can potentially facilitate the rapid recombination of the electrons and Li^+^. This, in turn, can foster the growth of metallic lithium and promote the localized nucleation of dendrites.^47^ As a result, reducing the overall electronic conductivity of SEs while preserving their high ionic conductivity poses a significant challenge to the successful advancement of ASSLMBs. Additionally, reduced electronic conductivity mitigates electron transfer at the electrolyte and lithium metal interface. This reduction in electron transfer helps to minimize the formation of thick SEI layers resulting from undesired side reactions at the interface.^14^ The electronic conductivities of *x*%-LPSC were assessed through direct-current polarization at 0.35, 0.7, 1.05, 1.4, and 1.45 V, given in Figure S5. The electronic conductivities of *x*%-LPSC at 0.35 V show 4.18 × 10^–8^, 1.47 × 10^–8^, 5.63 × 10^–9^, 4.34 × 10^–9^, 3.05 × 10^–9^, and 4.18 × 10^–10^ S cm^–1^ for *x* = 0, 1, 2, 3, 5, and 10 wt %, respectively, and the comparison of those values is given in Figure 3b. Therefore, the electronic conductivity value decreases with the increase in the PEG polymer, as shown in the current response graphs at different applied voltages. This decrease in electronic conductivity plays a role in curbing the nucleation and growth of lithium dendrite in the electrolyte, thereby preventing cracking and short-circuiting.^33^

The mechanical strength of the SE is directly linked to safety concerns because it determines its ability to withstand internal and external stresses during cell operation cycling.^48,49^ The mechanical strength test was performed on the 0%-LPSC and 3%-LPSC SE samples, and their stress–strain curves are presented in Figure 3d. The 3%-LPSC (1.18 N mm^–2^) SE exhibits higher tensile strength than the 0%-LPSC (0.83 N mm^–2^) SE, which is eligible for suppressing the growth of the Li dendrite. Furthermore, the strain in the 0%-LPSC SE measures 4.07%, whereas in the 3%-LPSC SE, it reaches 7.5%. This implies that including PEG enhances the tensile strength by addressing the weak particle–particle adhesion among the LPSC SE particles, resulting in a denser SE pellet. Additionally, it nearly doubles the ductility of the SE, leading to enhanced physical contact between the LPSC SE and the Li anode.

The cycling performances of symmetric cells with a Li|*x*%-LPSC|Li configuration were compared to assess the prolonged stability of Li plating/stripping, which is contingent on the deposition and growth of Li dendrites, as well as the interfacial degradation at the Li/SE interface. Consequently, it offers valuable insights into the general interfacial compatibility of the Li|*x*%-LPSC SE. The cells were subjected to cycling at a current density of 0.1 mA cm^–2^ under room-temperature conditions. As revealed in Figure 4a, the results showed that the Li|0%-LPSC|Li cell could only maintain stable charging/discharging for 380 h, after which a sudden voltage drop occurred, indicating complete short-circuiting. This observation indicates the presence of substantial parasitic reactions occurring between the SE and the Li anode, resulting in the formation of dendrites. Consequently, the dendrites exhibit a nonzero resistance, contributing to the sudden voltage drop and resulting in a short circuit.^50^ Moreover, the decomposition of LPSC electrolyte and the subsequent formation of an SEI consisting of Li_3_P, Li_2_S, and LiCl at the interface with the Li metal can contribute to a rise in interfacial impedance.^45,51^ This elevated impedance can lead to uneven plating of Li on the Li anode, ultimately shortening the cycle life of the 0%-LPSC SE.

Conversely, the Li|3%-LPSC|Li symmetric cell configuration demonstrated notably stable profiles of lithium plating and stripping for more than 3000 h without experiencing any short circuits (as depicted in Figure 4a). This observation indicates high stability in the interface between lithium and the 3%-LPSC SE, enabling effective facilitation of reversible lithium plating and stripping. The observed stability in the plating and stripping behavior within the 3%-LPSC SE can be attributed to various factors. First, the presence of the PEG polymer serves as a protective layer, effectively preventing direct contact between the SE and the Li metal anode. This protective barrier helps to mitigate the potential reactivity between the SE and Li metal. Second, the PEG polymer also reduces the nucleation and growth of Li dendrites within the SE by decreasing their electronic conductivity. This effect helps to repress the formation of dendritic structures, which can cause performance degradation and safety concerns. Additionally, the PEG polymer fills the voids among the sulfide particles, thereby enhancing the SE pellet’s compactness. This, in turn, enhances its mechanical strength, thereby preventing the penetration of the dendrite toward the SEs and promoting better contact between the SE and the Li metal anode by enhancing its ductility. Moreover, when in contact with the Li metal anode, the added LiFSI decomposes, creating an SEI enriched by LiF. Figure S6 displays an expanded galvanostatic symmetric cell voltage profile in the Li|3%-LPSC|Li configuration, recorded at different cycling times. On the other hand, EIS analysis was conducted on Li|Li symmetric cells before and after 50 cycles at 0.1 mA cm^–2^. Figure S7a,b illustrates the EIS spectra for symmetric cells with 0%-LPSC and 3%-LPSC SE, both before and after cycling, respectively. The graph indicates that, before cycling, the cell with the 3%-LPSC SE exhibited higher impedance, which can be attributed to its lower ionic conductivity. After cycling, the cell using the 0%-LPSC SE showed increased impedance. This rise in impedance is likely due to the reaction between 0%-LPSC and Li, leading to the decomposition of LPSC electrolyte and the subsequent formation of an SEI consisting of Li_3_P, Li_2_S, and LiCl at the interface.^45,51^ In contrast, the cell with the 3%-LPSC SE demonstrated a minimal increase in impedance, suggesting an enhancement in the stability of the Li and 3%-LPSC SE interface. This improvement can be attributed to the synergetic effect of the CSE and the in-situ-formed LiF-rich SEI. Furthermore, a Li|3%-LPSC|Li symmetric cell was assembled and subjected to testing at current densities of 0.5 (0.25 mA h cm^–2^ capacity), 1 (0.5 mA h cm^–2^ capacity), and 2 (1 mA h cm^–2^ capacity) mA cm^–2^ for 25, 30, and 65 cycles, respectively (Figure 4b). The cell demonstrated stable cycling at these tested current densities, suggesting that the designed CSE, along with the in-situ-generated LiF-rich SEI, contributes to outstanding stability.

Despite exhibiting enhanced ionic conductivity and reduced polarization in the Li|Li symmetric cell, 1 and 2% LPSC SEs have poor cyclability, indicating low interfacial stability. The observed phenomenon can be attributed to the possibility that the 1 and 2% polymer content may not be adequate to cover all LPSC particles fully and fails to fully protect the SEs from direct contact with the Li metal. This deficiency potentially leads to the decomposition of the LPSC electrolyte and an increase in interfacial resistance. Additionally, the in situ LiF SEI formed on the Li anode may not be sufficiently strong to guarantee homogeneous lithium deposition and inhibit dendrite growth as depicted in Figure S8 from the Li|Li symmetric test. Even though the Li|5%-LPSC|Li symmetric cell shows good cyclability, it has a higher voltage polarization when compared with the 3%-LPSC cell. Conversely, increasing the polymer content resulted in a gradual rise in overpotential from 10 mV (0%-LPSC) to 50 mV (10%-LPSC), as illustrated in Figure S8. This rise in the overpotential can be attributed to the decrease in ionic conductivity, as indicated in Figure 3a. This may adversely affect the high-rate capability of the cell. In summary, the 3%-LPSC composite SSE exhibited an optimized composition for balance between ionic conductivity, overpotential, and cycling life.

Another crucial parameter used to assess the interfacial stability and the ability of SEs to suppress dendrite formation is the CCD.^19^ It is the highest current density at which the cell can operate before experiencing a short-circuit event.^19,32,52^ It indicates the SE’s ability to withstand high current densities without dendrite growth or other detrimental effects. Suppose that the voltage abruptly declines to nearly zero at a particular current density during the gradual increase. In that case, this indicates a short circuit within the SE. This abrupt voltage drop suggests that Li dendrites have penetrated the SE, resulting in a short circuit.^38^ The CCD for 0%-LPSC and 3%-LPSC SEs was obtained by galvanostatic cycling on Li–Li symmetric cells (Figure 4c,d). The cells underwent a series of stepwise increases in current density. The 3%-LPSC SE exhibits a significantly higher measured CCD value of 4.75 mA cm^–2^ compared to the 0%-LPSC SE, which has a lower CCD value of 0.75 mA cm^–2^.

**Figure 5:**
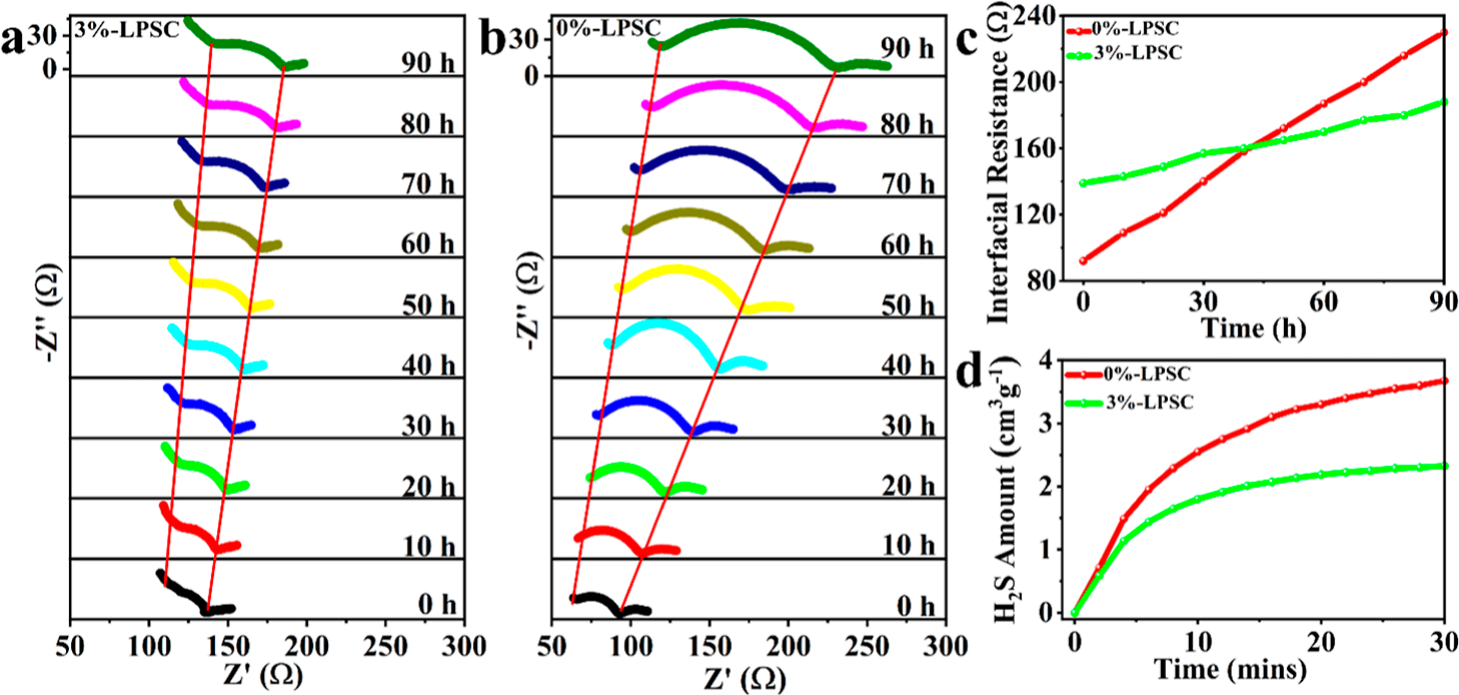
EIS spectra of (a) 3%-LPSC and (b) 0%-LPSC Li|SE|Li symmetric cells at various storage times; (c) time evolution resistance of Li|*x*%-LPSC|Li, where *x* = 0 and 3; (d) H_2_S amount generated at 20% moisture exposure for 0%-LPSC and 3%-LPSC SEs.

To further elucidate the extent of compatibility of the 3%-LPSC with the Li metal anode, interphase resistance growth at different storage times (0, 10, 20, 30, 40, 50, 60, 70, 80, and 90 h) was determined and compared with that of the 0%-LPSC SE. As depicted in Figure 5a, the symmetric cell with 3%-LPSC revealed slightly higher initial impedance compared to the cell with 0%-LPSC, which was primarily attributed to the lower ionic conductivity of the 3%-LPSC than that of the 0%-LPSC SEs. However, with a prolonged standing time, the impedance of the cell with 3%-LPSC electrolyte exhibited a slow increase, whereas the impedance of the cell with 0%-LPSC electrolyte increased rapidly (Figure 5b). As the storage time increased, the interphase impedance of the Li|0%-LPSC|Li cell increased from 90 to 230 Ω and that of the 3%-LPSC increased from 139 to 188 Ω only in 90 h. One possible reason for the higher increase in impedance for the 0%-LPSC system is the undesired side reaction between the LPSC SE and the Li metal anode.^18^ These reactions led to passivation layers, which function as barriers that hinder the mobility of Li^+^ ions, consequently causing the impedance to rise. This suggests that the 0%-LPSC SE is not compatible with the lithium metal anode, resulting in poor performance. On the other hand, the 3%-LPSC SE shows only a slight increase in impedance, indicating better compatibility with the lithium metal anode.^53^ The compatibility observed can be ascribed to the formation of a LiF-rich SEI layer, which is generated through the decomposition of LiFSI. In addition, the PEG additive contributes to this compatibility by reducing the electronic conductivity, enhancing the mechanical strength, serving as a barrier between the Li metal anode and the LPSC SE, and improving the contact between Li and the SE. The LiF-rich SEI layer helps suppress harmful parasitic reactions and improves the overall stability and electrochemical performance of the 3%-LPSC system. This observation is supported by XPS data, confirming the LiF compound’s occurrence in the SEI layer. Figure 5c presents a comparison of the growth in interfacial resistance between 3%-LPSC and 0%-LPSC SEs.

On the other hand, the moisture stability of 0%-LPSC and 3%-LPSC was tested with 20% relative humidity based on the amount of H_2_S evolved during a 20% air exposure as a function of time. As shown in Figure 5d, the 3%-LPSC releases less H_2_S when compared to the 0%-LPSC. This can be attributed to the presence of PEG polymer, which acts as a protective layer by enveloping the surface of LPSC, thus separating it from moisture and improving its humidity stability.

Additionally, cyclic voltammetry (CV) measurements were conducted on 3%-LPSC SE using Li as the reference electrode and SUS as the working electrodes to determine if the inclusion of PEG/LiFSI would impact the electrochemical stability window of the SE. The measurement was done with a 0.1 mV s^–1^ scan rate and from −0.5 to 5 V range vs Li/Li^+^. Figure S9 illustrates that the deposition of lithium (Li/Li^+^) is solely an observable peak at around 0 V, with no additional peaks observed. It suggests the as-prepared composite SE has a similar electrochemical window with the 0%-LPSC SE.^54^

**Figure 6:**
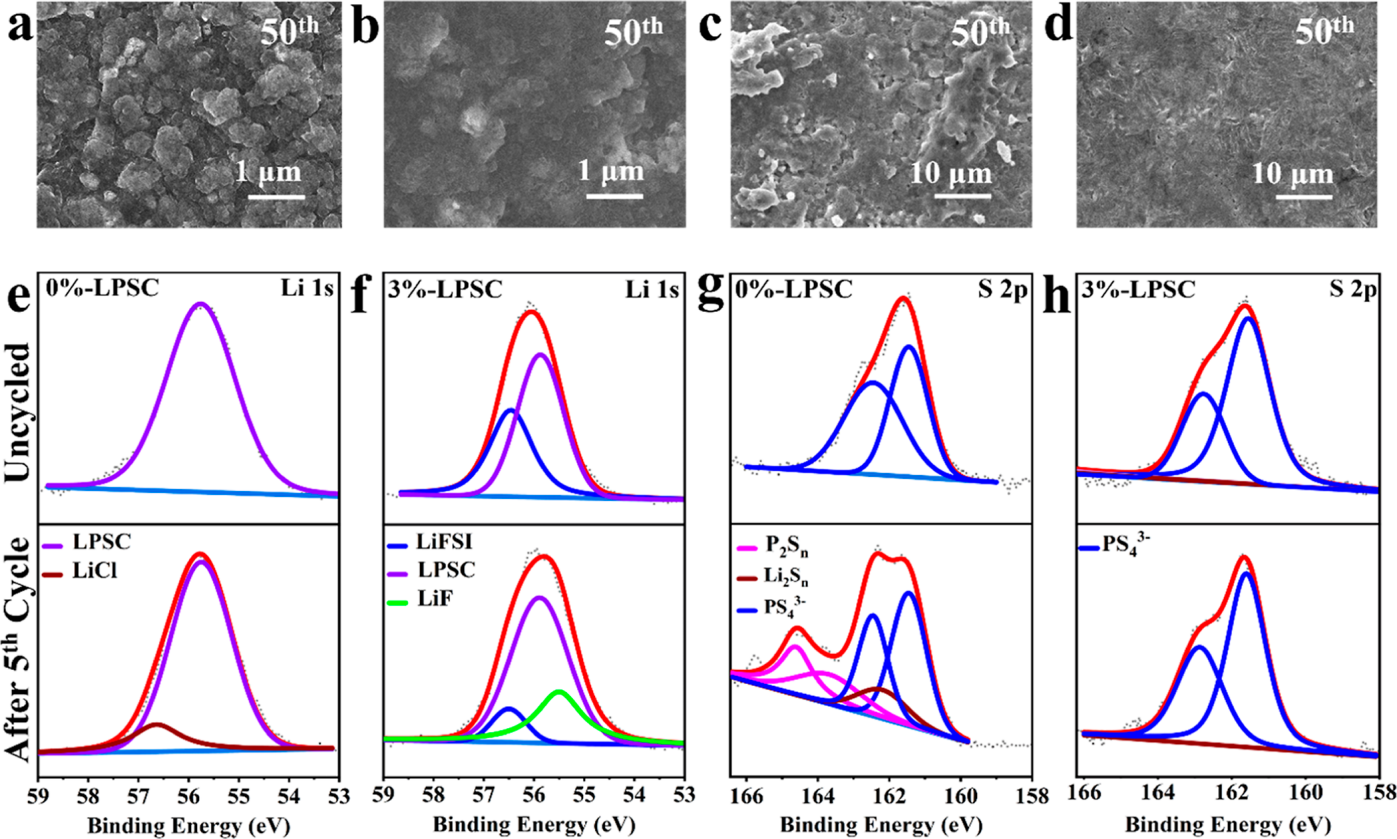
SEM images after the 50^th^ cycle; Li metal anode cycled with (a) 0%-LPSC and (b) 3%-LPSC and pellets of (c) 0%-LPSC and (d) 3%-LPSC SEs. XPS measurements on the pellet side before and after the 5^th^ cycle: (e) Li (1 s) and (g) S (2p) spectra for 0%-LPSC and (f) Li (1s) and (h) S (2p) spectra for 3%-LPSC.

To examine the surface morphology of the Li anode before and after cycling, SEM analysis was conducted. For the post cycling test, the lithium metal anodes were disassembled from the cells that had undergone cycling for 5 and 50 cycles at 0.1 mA h cm^–2^ areal capacity and 0.1 mA cm^–2^ current density. The uneven and porous surface observed on the lithium retrieved from the symmetric Li cell with 0%-LPSC (see Figure S10d for the 5^th^ cycle and Figure 6a for the 50^th^ cycle) is likely attributed to the reaction between the SE and the Li metal. This reaction can lead to inadequate interface contact and the creation of Li dendritic nucleation sites, which in turn can result in the formation and growth of Li dendrites. As the cycling process continues, it is well known that the thickness of this porous layer tends to increase, eventually failing the ASSLMBs.^10^ Furthermore, the surface morphology variations in lithium metal can result in an uneven distribution of current densities during charge and discharge cycles. This can eventually trigger the creation of high-surface-area lithium throughout charging and voids during discharging. Thus, voids generated at the interface of the lithium metal electrode during stripping weaken the interfacial contact and contribute to an increase in the cells’ overpotential. These high-surface-area lithium formations can exhibit a dendritic morphology in the most severe instances.

On the contrary, the surface of the lithium anode in contact with the 3%-LPSC CSE remains smooth even after cycling, as depicted in Figure S10b for the 5^th^ cycle and Figure 6b for the 50^th^ cycle. This observation indicates that the in situ generation of LiF offers effective protection against the interfacial reaction between LPSC and lithium metal. Furthermore, this in-situ-formed LiF-rich SEI facilitates homogeneous Li^+^ transport by reducing local current density and providing a stable scaffold. This stable scaffold promotes uniform electrodeposition/dissolution processes and effectively suppresses dendrite formation. Additionally, the in-situ-generated SEI layer exhibits several desirable properties such as (1) it serves as an excellent electronic insulator with a wide band gap, effectively preventing electron tunneling, and (2) it possesses high ionic conductivity, low diffusion energy, and high surface energy. These properties not only boost the rapid transport of Li^+^ but also promote the horizontal electrodeposition of Li rather than a vertical growth pattern.^55^

The surface morphology of the cycled SE pellets obtained from the symmetric cells Li|0%-LPSC|Li and Li|3%-LPSC|Li was also studied after the 5^th^ and 50^th^ cycles. After prolonged cycling, the recovered 0%-LPSC SE pellet exhibits significant cracking, as depicted in Figures S10c and 6c for the 5^th^ and 50^th^ cycles, respectively. This is due to the side reactions between the Li metal and the LPSC SE. Conversely, the SE pellet recovered from the cycled Li|3%-LPSC|Li cell (see Figure S10a for the 5^th^ cycle and Figure 6d for the 50^th^ cycle) exhibits a compact and undamaged surface without any signs of cracking, indicating higher resistance to such side reactions.

The Li|SE interface was analyzed using XPS, to evaluate the thermodynamic stability of 0%-LPSC and 3%-LPSC SEs against lithium metal and to examine the hypothesis of the in situ generation of LiF SEI. For this analysis, both uncycled and cycled pellets were utilized. In the case of the cycled pellet, the surface facing the Li electrode was specifically analyzed. The cycled pellet was obtained by disassembling the Li|Li symmetric cell at 0.1 mA cm^–2^ after the 5th cycle. XPS spectra of various elements including Li 1s, F 1s, P 2p, Cl 2p, and S 2p were collected.

The Li 1s spectrum of the uncycled 0%-LPSC (Figure 6e) surface exhibits a solitary peak at 56 eV, attributed to Li-thiophosphate. However, after cycling, its Li 1s spectra exhibit two separate peaks at 55.75 and 56.64 eV, which can be attributed to Li_6_PS_5_Cl and LiCl, respectively.^32,56^ Hence, the presence of a peak corresponding to LiCl, one of the potential decomposition byproducts of LPSC (as shown in eq 3), signifies the instability of the LPSC SE with a Li anode. This instability ultimately results in an elevated interfacial resistance, as evidenced by the impedance tests conducted at various storage times (Figure 5b) and the EIS measurement after cycling (Figure S7) for the Li|Li symmetric cell. In comparison, the Li 1s spectra of the 3%-LPSC SE exhibit two distinct peaks corresponding to LiFSI (56.5 eV)^57^ and LPSC (55.75 eV), which are common compositions observed in both the uncycled and cycled SEs, as depicted in Figure 6f. The cycled 3%-LPSC SE has one additional peak at 55.55 eV, corresponding to the LiF^57^ generated from the decomposition product of LiFSI. The observed evolution strongly supports the in situ formation of a LiF-rich SEI from the developed sulfide composite electrolyte. LiF is well known to be advantageous in forming a stable and compact SEI layer, which helps to stabilize the Li anode. The high interfacial energy of LiF (73.28 meV Å^–2^) serves a dual purpose: it promotes the diffusion of Li^+^ transport along the Li/LiF interface and concurrently reduces interfacial stress, promoting uniform Li deposition.^38,58^ Additionally, owing to its superior electronic insulation properties (approximately 10^–31^ S cm^–1^), LiF is highly effective at impeding the flow of electrons through the SEI layer.^59,60^

Regarding the F 1s spectra (Figure S11c), it is evident that the uncycled 3%-LPSC exhibits only one peak originating from the LiFSI salt added, located around 686.5 eV. Conversely, two signals are detected in the F 1s spectrum of the cycled 3%-LPSC SE. The signal at 683.5 eV can be ascribed to LiF, a decomposition product of LiFSI.^36^ The second signal observed at 686.45 eV is derived from the −S–F group present in LiFSI. This further confirms the in situ formation of a LiF-rich SEI.
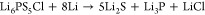
3

In the S 2p spectrum of the cycled and uncycled 3%-LPSC (as shown in Figure 6h), a sole doublet peak is evident at 161.45 eV. This peak is assigned to the PS_4_^3–^ tetrahedra within the LPSC argyrodite structure, corresponding to the −P–S–Li bonds.^10,61^ On the other hand, in the S 2p spectrum of the uncycled 0%-LPSC SE (Figure 6g), only one doublet peak is observed at 161.45 eV, which corresponds to the PS_4_^3–^ component of the LPSC.^10,36,61^ However, two additional peaks are observed after cycling, in addition to the doublet peak corresponding to PS_4_^3–^ of the LPSC. The doublet peak at 164.65 eV is attributed to phosphorus polysulfide (P_2_S_*n*_), while the singlet peak at 162.3 eV corresponds to lithium polysulfide (Li_2_S_*n*_). These peaks arise from the decomposition products of the LPSC SE during the cycling process, as given by eq 3,^51^ as a result of the instability of the LPSC SE with the Li metal anode. However, the characteristic peaks of Li_2_S_*n*_ (162.3 eV) and P_2_S_*n*_ (164.65 eV) observed in the case of 0%-LPSC SE, which are believed to be associated with the decomposition product of LPSC, are not detected in the 3%-LPSC sample. The absence of those peaks indicates the effective protection of the 3%-LPSC SE against the decomposition due to the interfacial reaction of the LPSC SE with the Li metal anode. This protection is achieved through the synergistic effect of the in-situ-formed LiF-rich SEI and the PEG additive. Therefore, introducing PEG/LiFSI additives can realize even lithium deposition and adjust the current density distribution, thereby mitigating the probability of interfacial reactions and dendrite growth. The XPS surface composition investigation substantiates substantial parasitic reactions between the LPSC SE and the Li metal during the Li stripping/plating process. This finding aligns with prior research findings.^10,32,51,56^ Additionally, the XPS spectra of Cl 2p and P 2p for both 0%-LPSC and 3%-LPSC SEs and the S 2p of the LiFSI are given in Figure S11.

**Figure 7:**
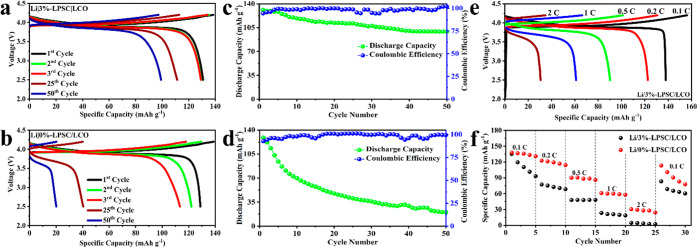
Electrochemical and rate performance of Li|*x*%-LPSC|LCO at 0.2 C rate. Charge/discharge curves in different cycles for (a) Li|3%-LPSC|LCO and (b) Li|0%-LPSC|LCO. The cycling performance of the cell for (c) the Li|3%-LPSC|LCO system and (d) the Li|0%-LPSC|LCO system. (e) Charge–discharge curves of the Li|3%-LPSC|LCO cell under different current densities. (f) Rate performance of Li|3%-LPSC|LCO and Li|0%-LPSC|LCO at various current densities from 0.1 to 2 C.

Galvanostatic charge–discharge tests were performed on full cells at a temperature of 30 °C, from 2.5 to 4.2 V voltage range. The tests were performed at a 0.2 C rate, where 1 C corresponds to 140 mA h g^–1^ (∼1.25 mA h cm^–2^). Figure 7 presents the electrochemical and rate performance data of Li|3%-LPSC|LCO and Li|0%-LPSC|LCO ASSLMBs. In Figure 7a, the Li|3%-LPSC|LCO cell exhibits a higher rate of curve overlap during the initial cycles. This indicates better cycling stability and consistent capacity retention. On the other hand, in Figure 7b, the Li|0%-LPSC|LCO cell shows continuous capacity decay over time. This decay is attributed to active lithium consumption and the development of an unstable SEI layer, which has a detrimental effect on the cyclability of the cell. The Li|3%-LPSC|LCO cell demonstrates a specific capacity of 130.85 mA h g^–1^ (∼1.17 mA h cm^–2^) during the first discharge. Additionally, it exhibits an initial Coulombic efficiency of 94.48%. After 50 cycles, the discharge capacity of the Li|3%-LPSC|LCO cell remains approximately 99.15 mA h g^–1^ (∼0.88 mA h cm^–2^), resulting in a retention rate of 75.77%. On the other hand, the Li|0%-LPSC|LCO cell retains only a discharge capacity of 20.17 mA h g^–1^ (∼0.18 mA h cm^–2^) after 50 cycles, equating to a capacity retention of 15.66%. The modified sample, Li|3%-LPSC|LCO, exhibits a substantial enhancement in capacity retention of approximately 60.11% (nearly five times higher than the 0%-LPSC SE). On the other hand, the EIS results before and after cycling (Figure S12a,b), comparing Li|3%-LPSC|LCO and Li|0%-LPSC|LCO batteries, provide insights into the enhanced cycling performance of ASSBs incorporating the 3%-LPSC SE. After 25 cycles at 0.2 C, it is evident that the cell utilizing the 3%-LPSC SE exhibits minimal impedance increase compared to its precycling state. This observation highlights the effective stabilization of the interface between the Li metal anode and the 3%-LPSC SE, attributed to the synergistic effects of the CSE and the in-situ-formed LiF-rich SEI. In contrast, the cell utilizing the 0%-LPSC SE exhibits a rapid increase in impedance after cycling, and the EIS results support the superior electrochemical performances of the LCO|3%-LPSC|Li batteries.

Moreover, Figure 7e presents the charge–discharge curves of the Li|3%-LPSC|LCO cell under various current densities, while the rate performances of both the Li|0%-LPSC|LCO and Li|3%-LPSC|LCO cells are evaluated in Figure 7f. At rates of 0.1 0.2, 0.5, 1, and 2 C, the full cell utilizing the 3%-LPSC SE delivers specific capacities of 137.97, 122.61, 90.19, 61.05, and 30.57 mA h g^–1^, respectively. Upon reducing the current density back to 0.1 C, the specific capacity can recover to 113.19 mA h g^–1^. In contrast, the 0%-LPSC SE yields specific capacities of 135.35, 77.28, 47.98, 23.35, and 4.72 mA h g^–1^ at these corresponding currents. The diminished electrochemical performance of the 0%-LPSC SE is directly linked to a substantial rise in interfacial resistance (as illustrated in Figure S12) and interface degradation resulting from inadequate contact between the 0%-LPSC and lithium metal as well as uneven lithium deposition. Overall, the remarkable improvement in the rate and cycling performance of the Li|3%-LPSC|LCO battery can be ascribed to the synergistic effects of the CSE and the in situ formation of a LiF-rich SEI.

## 4 Conclusions

Our findings present a pioneering and simple approach to mitigating lithium dendrite growth compared with previous methods. This study demonstrates effective suppression of dendrite growth by in situ formation of a LiF-rich SEI and promoting a compliant contact between SSEs and the lithium surface. Our approach offers notable advantages. First, the solvent-free preparation of the solid sulfide composite electrolyte at room temperature eliminates the need for a solvent. Second, the in situ formation of LiF at the SSE–lithium interface provides exceptional electrochemical stability, mitigating the reduction of LPSC and enabling the horizontal deposition of Li. Lastly, our method is simple, cost-effective, easily scalable and suitable for large-scale production. The incorporation of PEG additives also brings additional benefits. It enhances sulfide particle adhesion, preventing dendrite penetration; it improves contact between the Li metal anode and SEs and addresses contact loss issues by increasing the ductility of the SE. Consequently, the Li|Li symmetric cell with the 3%-LPSC SE exhibits impressive cycling stability without shorting over 3000 h under 0.1 mA cm^–2^ and 0.1 mA h cm^–2^ at room temperature. Furthermore, the Li|3%-LPSC|LCO ASSLMB shows higher capacity retention and high-rate performance than the Li|0%-LPSC|LCO ASSLMB. Our approach offers a new choice for sulfide-based composite SE synthesis and the strategy for protecting lithium anodes using an in situ LiF-rich interface layer, demonstrating the promising potential for utilizing lithium anodes in ASSBs.

## Supporting Information Available

EDS elemental mapping of the 3%-LPSC CSE, XRD patterns of PEG and LiFSI, structural characterization of the prepared CSE by XRD and Raman spectroscopy after 45 days of storage time, Nyquist plot for temperature-dependent EIS measurement of 0%-LPSC and 3%-LPSC SEs from 25 to 85 °C, current responses at different applied voltages as a function of time for *x*%-LPSC SEs, galvanostatic symmetric voltage profiles of Li|3%-LPSC|Li at 0.1 mA cm^–2^ current density and 0.1 mA h cm^–2^ at different cycling hours, EIS spectra for Li|Li symmetric cells before and after cycling, galvanostatic Li plating/stripping profiles of the Li|*x*%-LPSC|Li symmetric cell at 0.1 mA cm^–2^, electrochemical stability test of the 3%-LPSC SEs by CV, SEM images after the 5th cycle of both 3%-LPSC and 0%-LPSC pellets, as well as the Li metal anode cycled with each of the SEs, XPS spectra at Li|SE interface chemical composition test on the pellet side before and after the 5th cycle for both 0%-LPSC and 3%-LPSC SEs, and EIS spectra for Li|*x*%-LPSC|LCO before cycling and after the 30th cycle (PDF)
