# Supplementary material for: Boosting the Interfacial Stability of the Li6PS5Cl Electrolyte with a Li Anode via In Situ Formation of a LiF-Rich SEI Layer and a Ductile Sulfide Composite Solid Electrolyte
Source: ACS Appl Mater Interfaces. 2024 Feb 15;16(8):10832–44. doi: 10.1021/acsami.3c14763 (PMC10910511; doi:10.1021/acsami.3c14763)
Supplement: Supplementary file 1 — am3c14763_si_001.pdf [file am3c14763_si_001.pdf]

Supporting Information

Boosting the Interfacial Stability of Li_6PS_5Cl Electrolyte with Li Anode via In-situ Formation of LiF-rich SEI Layer and Ductile Sulfide Composite Solid Electrolyte

Gashahun Gobena Serbessa [a, c], Bereket Woldegbreal Taklu [b], Yosef Nikodimos [a], Nigusu Tiruneh Temesgen [a], Zabish Bilew Muche [a], Semaw Kebede Merso [a], Tsung-I Yeh [a], Ya-Jun Liu [a], Wei-Sheng Liao [b], Chia-Hsin Wang [e], She-Huang Wu [b, f, *], Wei-Nien Su [b, f, *], Chun-Chen Yang [c, d, *], Bing Joe Hwang [a, e, f, *]

[a] Nano-electrochemistry Laboratory, Department of Chemical Engineering, National Taiwan University of Science and Technology, Taipei City, 106, Taiwan

[b] Nano-electrochemistry Laboratory, Graduate Institute of Applied Science and Technology, National Taiwan University of Science and Technology, Taipei City, 106, Taiwan

[c] Battery Research Center of Green Energy, Ming-Chi University of Technology, New Taipei City, 24301, Taiwan

[d] Department of Chemical Engineering, Ming Chi University of Technology, New Taipei City, 24301, Taiwan

[e] National Synchrotron Radiation Research Center (NSRRC), Hsinchu, 30076, Taiwan

[f] Sustainable Electrochemical Energy Development (SEED) Center, National Taiwan University of Science and Technology, Taipei City, 106, Taiwan

Corresponding authors:

Bing Joe Hwang (bjh@mail.ntust.edu.tw)

Chun-Chen Yang (ccyang@ mail.mcut.edu.tw)

Wei-Nien Su (wsu@mail.ntust.edu.tw)

She-Huang Wu (wush@mail.ntust.edu.tw)

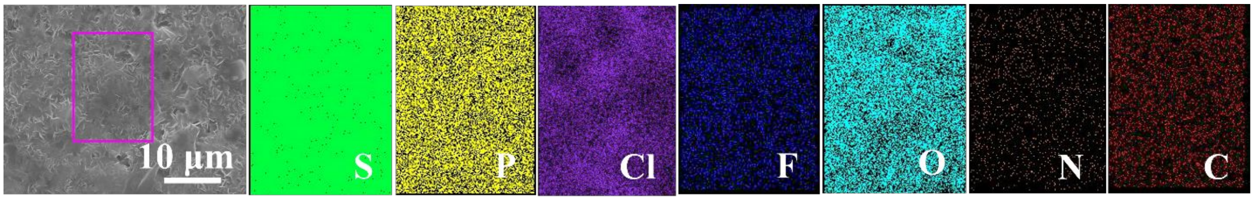

Figure S1. EDS elemental mapping of the 3%-LPSC composite solid electrolyte for S, P, Cl, F, O, N, and C elements, respectively.

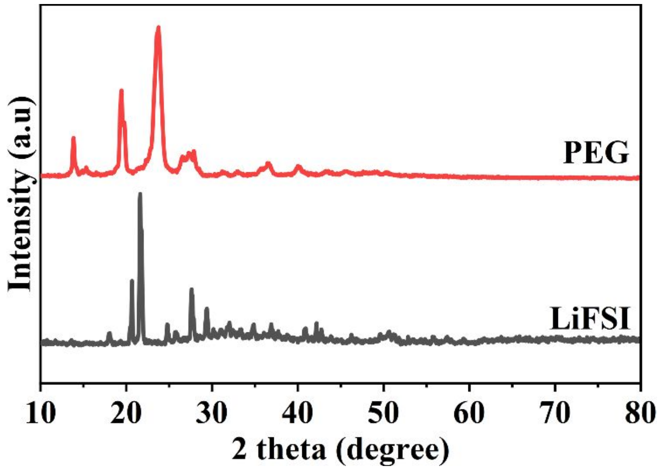

Figure S2. XRD patterns of PEG and LiFSI.

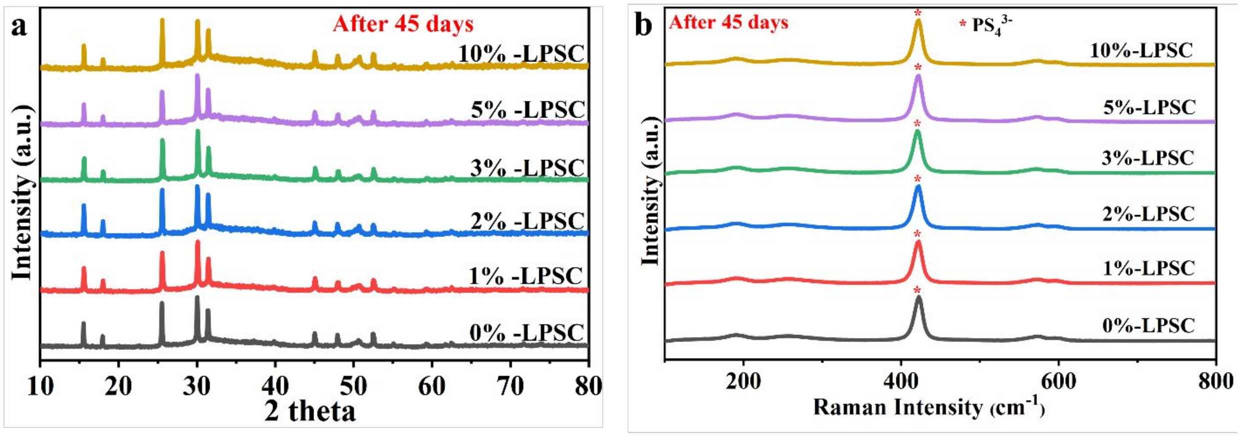

Figure S3. Structural characterization after 45 days storage time in the glove box a) XRD b) Raman spectra of the prepared composite solid electrolyte.

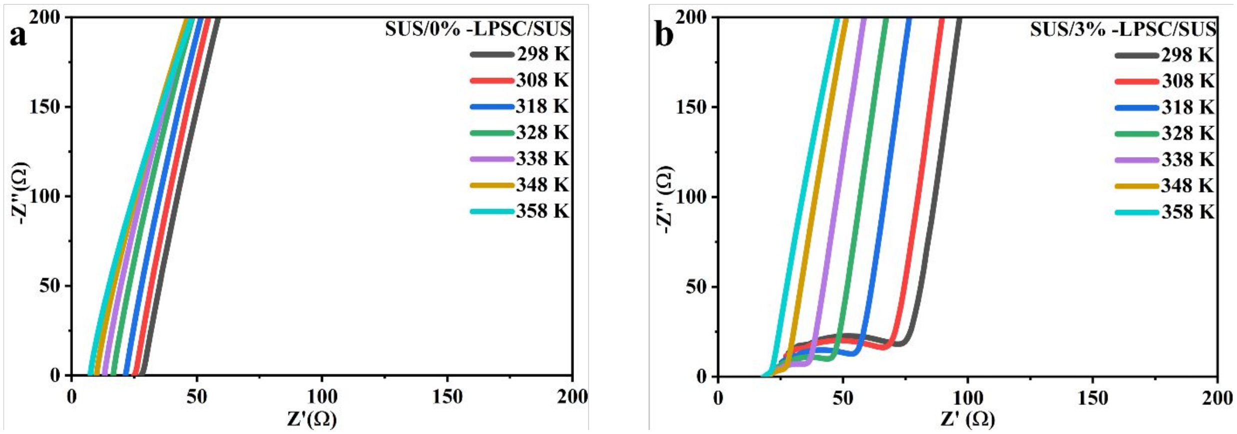

Figure S4. Nyquist plot for temperature-dependent EIS measurement of the 0%-LPSC (a) and 3%-LPSC (b) from 25 °C to 85 °C.

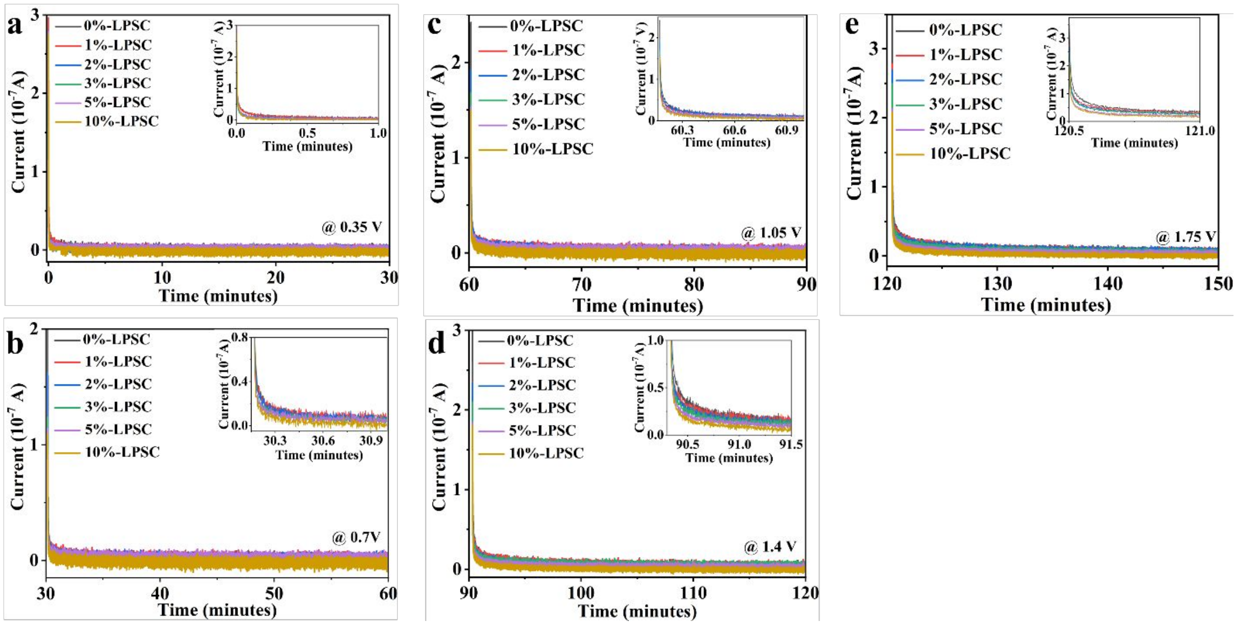

Figure S5. Current responses at different applied voltages as a function of time for the x%-LPSC (a-e) plots were collected at 0.35, 0.7, 1.05, 1.4, and 1.75 V, respectively.

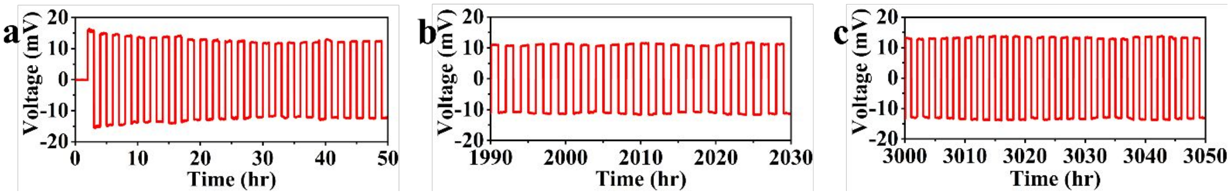

Figure S6. Galvanostatic symmetric voltage profiles of Li|3%-LPSC|Li at 0.1 mA cm^{-2} current density and 0.1 mAh cm^{-2} at RT, extracted from (a) 0-50 (b) 1990-2030 (c) 3000-3050 cycling hours.

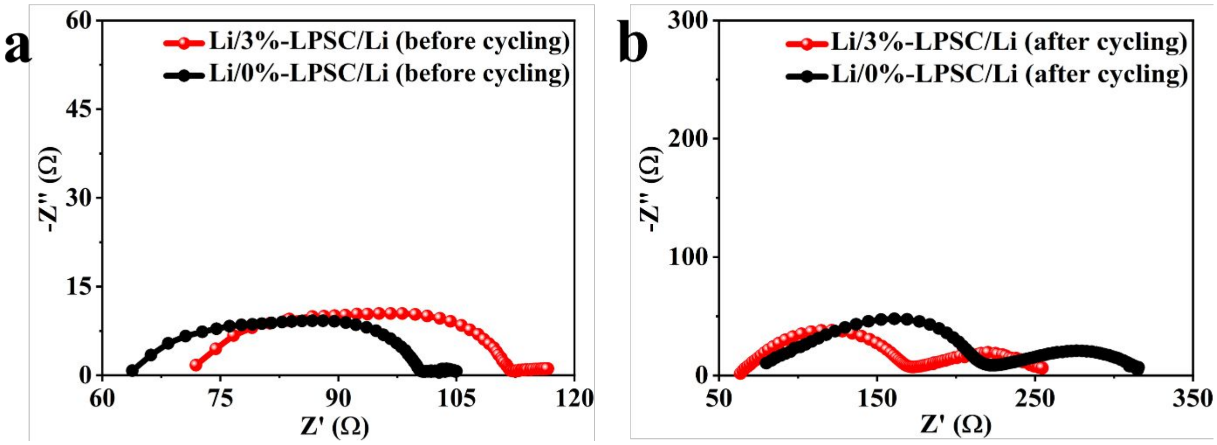

Figure S7. EISs for Li/Li symmetric cells were obtained (a) before cycling and (b) after 50 cycles.

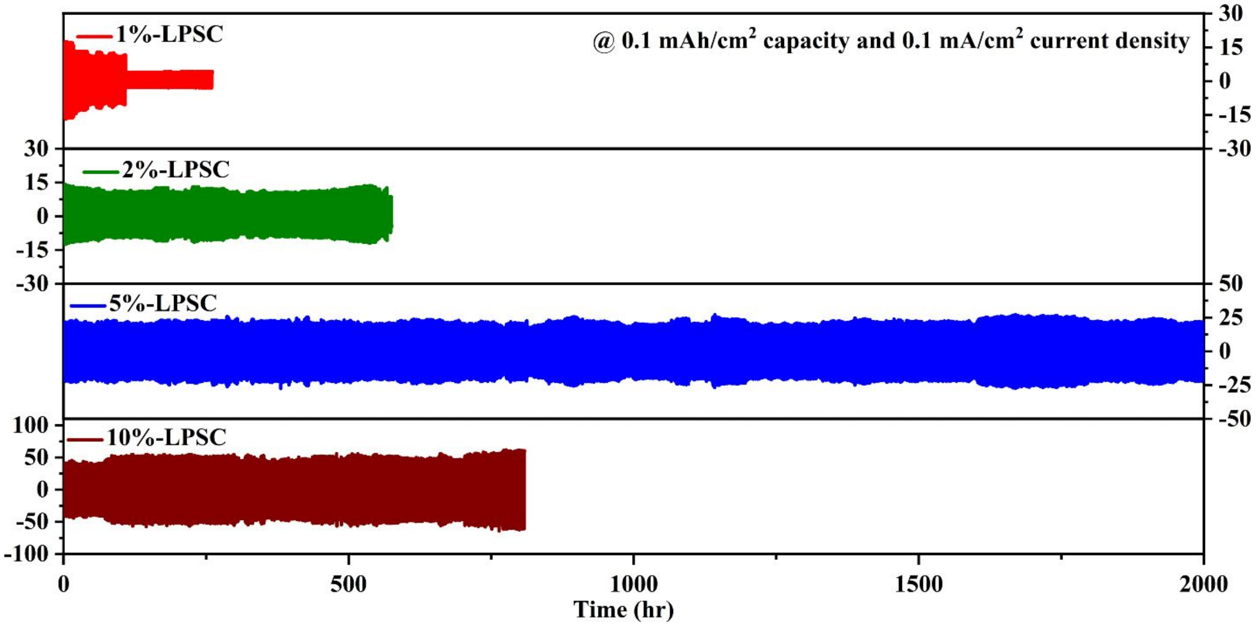

Figure S8. Galvanostatic Li plating/stripping profiles of Li/x%-LPSC/Li symmetric cell at 0.1 mA cm^{-2} under 25 °C, where x = 1, 2, 5, and 10.

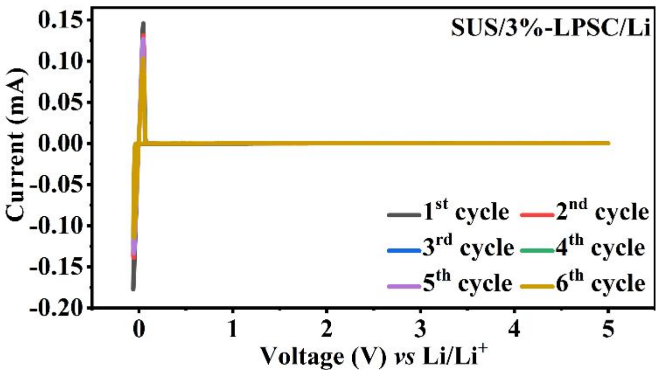

Figure S9. The electrochemical stability test of the 3%-LPSC solid electrolytes by cyclic voltammetry.

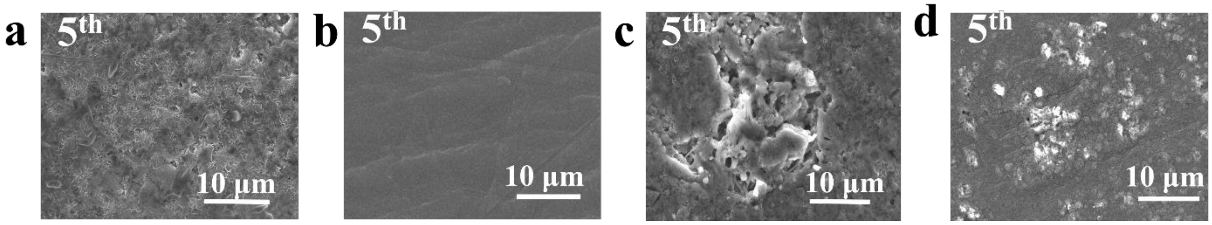

Figure S10. SEM images after cycling: (a) exhibit 3%-LPSC pellets after the 5th cycle; (b) showcase the Li metal anode cycled with 3%-LPSC after the 5th cycle; (c) display 0%-LPSC pellets after the 5th cycle; and (d) illustrate the Li metal anode cycled with 0%-LPSC after the 5th cycles.

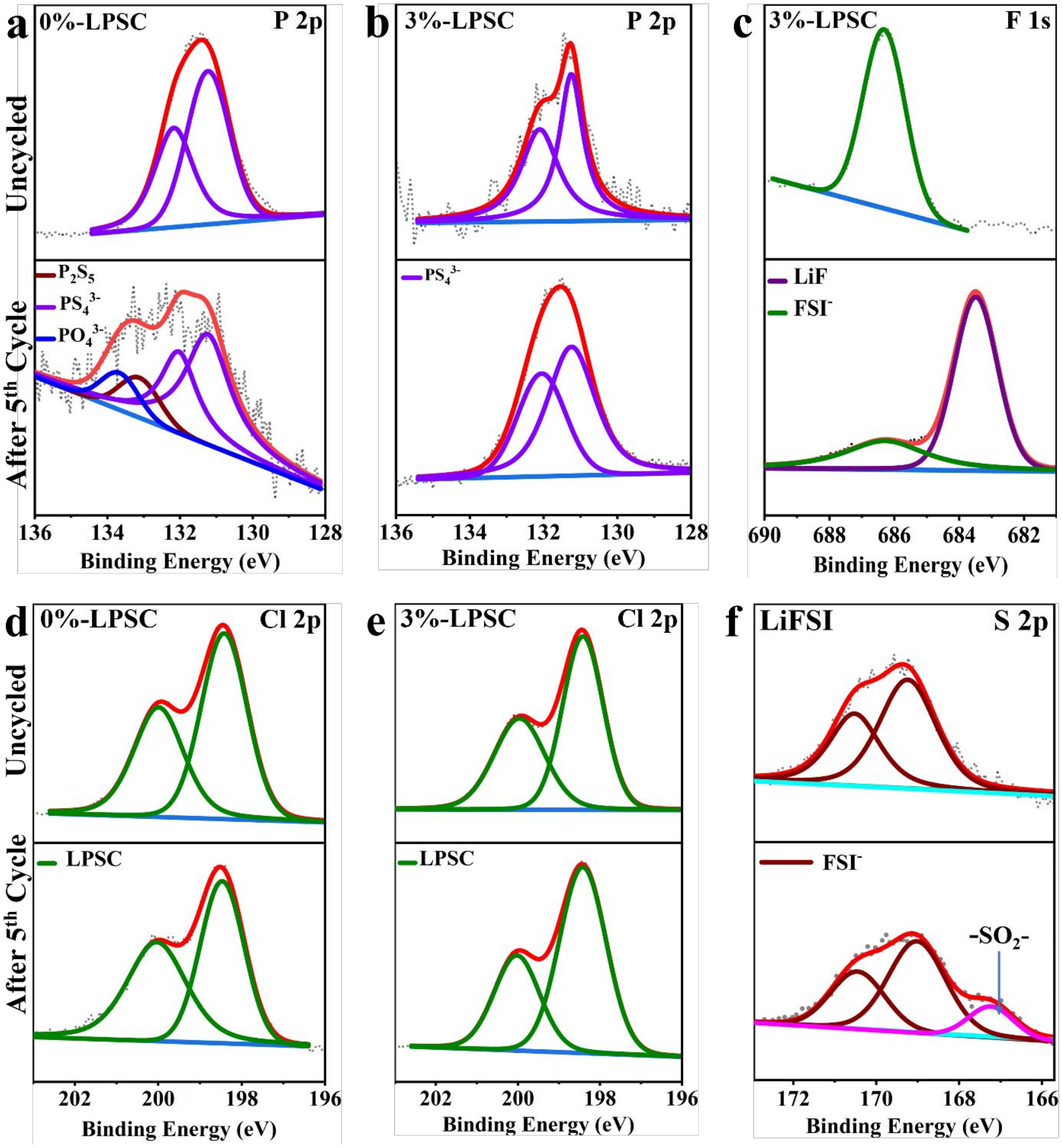

Figure S11. XPS spectra at Li/SE interface chemical composition test on the pellet side before and after the 5th cycle a) P (2p) and (d) Cl (2p) spectra for 0%-LPSC b) P (2p) (c) F (1s) (e) Cl (2p) spectra for 3%-LPSC, f) S (2p) of LiFSI respectively.

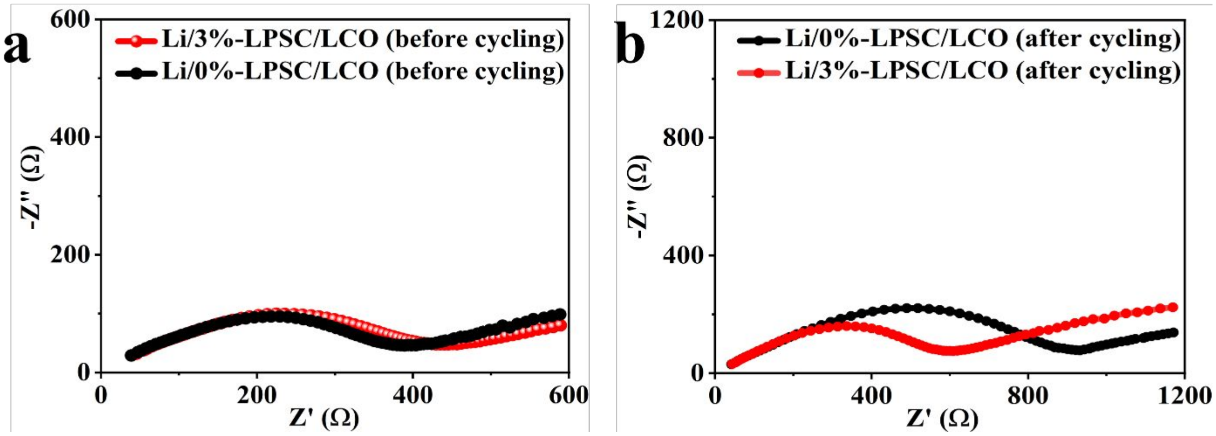

Figure S12. EISs for Li/x%-LPSC/LCO (c) before cycling, and (d) after the 30th cycle.
